# Cortical anatomical variations, gene expression profiles, and clinical phenotypes in patients with schizophrenia

**DOI:** 10.1016/j.nicl.2023.103451

**Published:** 2023-06-09

**Authors:** Yong Han, Yongfeng Yang, Zhilu Zhou, Xueyan Jin, Han Shi, Minglong Shao, Meng Song, Xi Su, Qi Wang, Qing Liu, Wenqiang Li, Luxian Lv

**Affiliations:** a Department of Psychiatry, Henan Mental Hospital, The Second Affiliated Hospital of Xinxiang Medical University, Xinxiang, China; b Henan Key Lab of Biological Psychiatry, International Joint Research Laboratory for Psychiatry and Neuroscience of Henan, Xinxiang Medical University, Xinxiang, China

**Keywords:** Magnetic resonance imaging, Morphology, Gene expression, Clinical phenotype, Schizophrenia

## Abstract

**Background and Hypothesis:**

Schizophrenia (SZ) patients display significant structural brain abnormalities; nevertheless, the genetic mechanisms regulating cortical anatomical variations and their correlation with the disease phenotype are still ambiguous.

**Study Design:**

We characterized anatomical variation using a surface-based method derived from structural magnetic resonance imaging of patients with SZ and age- and sex-matched healthy controls (HCs). Partial least-squares regression was performed across cortex regions between anatomical variation and average transcriptional profiles of SZ risk genes and all qualified genes from the Allen Human Brain Atlas. The morphological features of each brain region were correlated to symptomology variables in patients with SZ using partial correlation analysis.

**Study Results:**

A total of 203 SZ and 201 HCs were included in the final analysis. We observed significant variation of 55 regions of cortical thickness, 23 regions of volume, 7 regions of area, and 55 regions of local gyrification index (LGI) between SZ and HC groups. Expression profiles of 4 SZ risk genes and 96 genes from all qualified genes showed a correlation to anatomical variability, however, after multiple comparisons, the correlations were no longer significant. LGI variability in multiple frontal subregions was associated with specific symptoms of SZ, whereas cognitive function involving attention/vigilance was linked to LGI variability across nine brain regions.

**Conclusions:**

Cortical anatomical variation of patients with schizophrenia is associated with gene transcriptome profiles as well as clinical phenotypes.

## Highlights

• This study examined the relationship between cortical anatomical variation with gene expression and clinical phenotypes of schizophrenia patients.
• The anatomical features of the brain cortical in patients with schizophrenia exhibit widespread and consistent variation, which is associated with the transcriptome profiles of genes, especially cortical thickness and local gyrification index.
• Significant relationships found between cortical thickness and local gyrification index with symptomatology and cognitive function involving multiple brain regions of patients with schizophrenia.

## 1 Introduction

Schizophrenia is a severe mental illness that presents with symptoms such as hallucinations, delusions, cognitive impairment, and loss of initiative. It carries significant health risks, leading to reduced life expectancy, increased risk of suicide (Plana-Ripoll et al., 2019), and physical illnesses (Momen et al., 2020). The economic and social costs associated with schizophrenia are also substantial (Andreasen, 1991). While treatments can alleviate some symptoms, chronic symptoms may persist, and adverse side effects remain prevalent (Jaaskelainen et al., 2013). A lack of understanding of the underlying biological mechanisms has hindered the development of valid biomarkers and effective therapies for this disabling syndrome, making its treatment challenging (Singh and Rose, 2009/07/01 2009).

There is substantial evidence supporting a significant genetic contribution to the risk of developing schizophrenia (SZ). Family, twin, and adoption studies have estimated the heritability of this disorder to be between 70 and 80% (Kahn et al., 2015, St Clair and Lang, 2021/05/30/ 2021,). Recently, the Schizophrenia Working Group of the Psychiatric Genomics Consortium (PGC) conducted two genome-wide association studies (GWAS), which identified 108 and 287 common associated loci in 2014 (Biological insights from 108 schizophrenia-associated genetic loci, 2014) and 2022 (Trubetskoy et al., Apr 2022), respectively. Enrichment of common variant associations was restricted to genes expressed in excitatory and inhibitory neurons of the central nervous system, but not other tissues or cell types (Kathuria et al., 2020), indicating that neurons are the primary site of pathology in schizophrenia.

Individuals with SZ exhibit distinctive morphological abnormalities in the brain, and structural magnetic resonance imaging (s-MRI) has been widely used to study these changes (Bora et al., 2011, Amann et al., 2016, Haijma et al., 2012, Shepherd et al., 2012/04/01/ 2012,). Voxel-based morphometry (VBM) and surface-based morphometry (SBM) analysis methods are particularly relevant, as they allow the detection of focal changes in grey matter (GM) tissue. VBM can identify regional differences in GM volume, while SBM measures local features of the cortex, such as cortical thickness (CT), cortical area (CA), local gyrification index (LGI), and cortical volume (CV). These metrics reflect different aspects of cerebral cortical microstructure and may be associated with differential genetic and cellular mechanisms in the brain. Investigating these structural brain abnormalities in schizophrenia will contribute to our understanding of its etiology, progression, and treatment efficacy. Nonetheless, despite ongoing research, the structural changes and underlying biological mechanisms that give rise to schizophrenia remain incompletely understood, and there have been limited advancements in diagnosis and treatments.

A recent study has observed a strong genetic overlap between schizophrenia and brain morphology, with 20% of the loci and 50% of the genes significantly associated with schizophrenia also showing significant association with brain morphology (van der Meer et al., 2022). This finding points to the potential utility of incorporating genetic information on brain morphometry to enhance the power of schizophrenia genetic studies. By combining genomic and imaging data, it may be possible to develop a more integrated understanding of schizophrenia that can inform future treatments.

One promising direction for exploring genetic associations is to investigate brain imaging phenotypes in relation to transcriptional profiles (Romme et al., 2017, Roshchupkin et al., 2016, Ortiz-Terán et al., 2017, Xu et al., 2018). Romme et al. (Romme et al., 2017) examined the role of genes in brain connectivity among schizophrenia patients and found that regional dysconnectivity was significantly correlated with the expression profile of schizophrenia risk genes across cortical regions, particularly those related to neuronal calcium signaling. Genes that demonstrated high correlation were primarily involved in synapse formation and protein complexes (Romme et al., 2017). Similarly, Morgan et al. (Morgan et al., 2019) proposed an approach called morphometric similarity mapping that quantified the structural similarity between brain regions in individuals with psychosis. They found that this anatomical pattern correlated with the expression of genes associated with nervous system development, synaptic signaling, schizophrenia, and antipsychotic treatments.

To our knowledge, no studies have yet examined the correlation between surface-based cortical morphometric features, gene transcription profiles, and clinical phenotype in individuals with SZ. In this study, we used SBM to analyze CT, CA, CV, and LGI in patients with SZ and a control group of healthy individuals. Identifying specific morphological variations in SZ patients and elucidating their relationship with brain transcriptome and clinical measurements will shed light on the neurobiological determinants of SZ and provide valuable insights for managing and treating this complex disorder.

## 2 Methods and materials

### 2.1 Study design

**Fig. 1:**
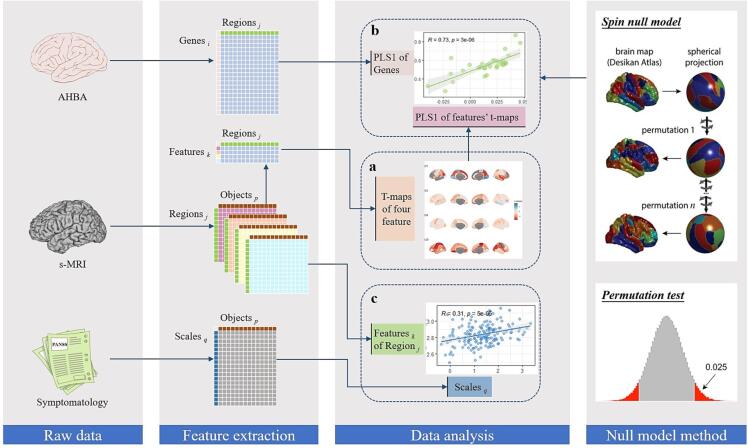
Overview of data processing and analysis. **(a)** The variance (t-map) of morphological variables between patients with schizophrenia and healthy controls was calculated; **(b)** the t-maps of features were correlated to schizophrenia risk genes and qualified genes from AHBA; **(c)** the link between morphological features of each brain region and symptomatology indicators was explored using partial correlation analysis; and the schematic diagram of the spatial spin null model as well as permutation test. AHBA: Allen Human Brain Atlas; s-MRI: Structural magnetic resonance imaging.

The aim of our study is to investigate whether cortical anatomical alterations in schizophrenia are underpinned by genetic factors and associated with clinical symptoms. First, we compared patients with schizophrenia to healthy controls to describe morphological variance and detect differences between the two groups. Next, we examined the relationship between SZ-related morphological variations and comprehensive expression levels of SZ risk genes extracted from the Allen Human Brain Atlas (AHBA) database (Sunkin et al., Jan 2013). Schizophrenia risk genes were derived from genes identified in two GWAS studies done by the Schizophrenia Working Group of the PGC. Then, we conducted an exploratory correlation analysis of all qualified genes in the AHBA dataset with SZ-related morphological variations. Finally, we executed correlation analyses involving morphological indices and clinical scale scores. The workflow of the analysis is illustrated in Fig. 1.

### 2.2 Subjects

A total of 404 participants were included in our study, consisting of 203 patients with schizophrenia (SZ) recruited from the inpatient department at the Second Affiliated Hospital of Xinxiang Medical University and 201 age- and gender-matched healthy controls (HCs) enrolled from communities of Xinxiang city. The Structured Clinical Interview for DSM-IV Axis I Disorders (SCID-I) patient version was used for the diagnosis of SZ, and two experienced psychiatric physicians reached consensus diagnoses. Patients were excluded when they (i) suffered from another diagnosed Axis I psychiatric disorder; (ii) had organic brain disorders, neurological disorders, or a serious physical illness; and (iii) were unable to undergo MRI scanning or had any metal or electronic implants. HCs were recruited using the following criteria: (i) not diagnosed with any mental illness according to the SCID-I nonpatient version, (ii) no history of psychiatric illness among their first-degree relatives, and (iii) no history of substance or alcohol dependence. After obtaining approval from the local ethics committee and providing all participants with a detailed description of the risks and benefits, written informed consent was obtained from each participant. Further details on participant characteristics can be found in Table 1. In addition, each patient underwent scale assessment including the Positive and Negative Syndrome Scale (PANSS) and MATRICS Consensus Cognitive Battery (MCCB) on the same day as their MRI scan.

**Table 1:** Demographics of schizophrenia patients and healthy control subjects.

Characteristics	SZ (N = 203)	HC (N = 201)	*t*/*χ*^2^	*p value*
Age, mean (SD), year	31.897±9.139	31.294±8.703	0.677	0.499
Sex, male/female	108/96	105/97	0.038	0.846
Educational level			47.065	< 0.001
(Unfinished) primary school	20	7		
Middle school	32	73		
High school	46	53		
Junior college	60	27		
University	45	29		
Postgraduate	11	1		
Duration of illness, mean ± SD, year	7.029±6.227 (n = 203) ^a^			
PANSS scores, mean ± SD				
Total score	92.683±13.825 (n = 162)			
Positive symptoms	24.469±6.240 (n = 162)			
Negative symptoms	25.080±5.999 (n = 162)			
General psychopathology symptoms	43.025±8.558 (n = 162)			
MCCB scores, mean ± SD				
Trail Making, Part A	69.096±36.472 (n = 188)			
BACS symbol coding	37.946±13.121 (n = 186)			
Category fluency	14.982±6.265 (n = 188)			
HVLT-R	16.218±6.552 (n = 189)			
WMS-III spatial span	11.550±3.734 (n = 182)			
NAB mazes	8.473±6.486 (n = 182)			
BVMT-R	16.945±10.395 (n = 188)			
CPT-IP	1.348±0.772 (n = 169)			

Note: PANSS = Positive and Negative Syndrome Scale; SD = standard deviation; MCCB = MATRICS Consensus Cognitive Battery; BACS = Brief Assessment of Cognition in Schizophrenia; WMS-III = Wechsler Memory Scale, Third Edition; NAB = Neuropsychological Assessment Battery; HVLT-R = Hopkins Verbal Learning Test-Revised; BVMT-R = Brief Visuospatial Memory Test-Revised; CPT-IP *=* Continuous Performance Test-Identical Pairs. a Different *n* are due to missing values on one or more tests for some subjects.

### 2.3 MRI data acquisition

MRI data was acquired using 3.0-T MR scanners (Siemens, Verio, Germany) by a skilled radiological technician. A standard scanning head coil was used, and all subjects were placed supine in the MRI machine. During the scan, subjects remained awake and motionless with eyes closed, without performing any cognitive task. The following acquisition parameters for T1-weighted scans were used: repetition time (TR)/echo time (TE) = 2,530/2.43 ms, echo time = 2.43 ms, inversion time = 1,100 ms, field of view (FOV) = 256×256 mm, matrix = 256×256, flip angle (FA) = 7°, layer thickness = 1 mm, isotropic voxel size = 1 mm^3^, no spacing, number of layers = 192, and scan time 480 s.

### 2.4 Estimation of morphological parameters

We employed FreeSurfer package 7.1.0 (https://surfer.nmr.mgh.harvard.edu) to conduct surface-based morphometry (SBM) analysis and obtain morphological measurements on the Ubuntu (version 12.04) platform. While complete technical details for the use of FreeSurfer are available elsewhere (Fischl, 2012), here, we succinctly describe an overview of the preprocessing procedure. Specifically, nonbrain structures such as skulls were removed and images registered to MNI305 space. The interface between grey matter and white matter (called the white surface), as well as regions containing grey matter and cerebrospinal fluid/dura (called the pial surface), were estimated in brain tissues. Thereafter, a 3D reconstruction of cortical surfaces was performed. We conducted data quality control by both visual inspection and calculating Euler values (Rosen et al., 2018), and the calculated Euler values of both hemispheres of all participants were 2.The Desikan-Killiany atlas (Desikan et al., 2006) was used to parcel each subject’s cortical model into distinct regions of interest (ROIs). Subsequently, we computed surface-based measures, specifically cortical thickness (CT), cortical area (CA), cortical volume (CV) and local gyrification index (LGI), at the ROI level for all participants.

### 2.5 Gene expression data processing

We utilized the AHBA transcriptomic dataset (https://www.brain-map.org) to obtain transcriptional profiles, which comprised 58,692 probes for 20,737 genes across the entire cortical mantle. The dataset was derived from six post-mortem brain samples donated by adults without any history of neurological or psychiatric ailments. Since only two donors had tissue samples of the right hemisphere, and six donors had tissue samples of the left hemisphere. Therefore, samples of the left hemisphere were selected for analysis. These data were pipelined using the ‘abagen’ toolbox that was developed to link whole-brain gene expression profiles to neuroimaging data (Markello et al., 2021). Briefly, we reannotated microarray probe-to-gene mappings with information from the latest NCBI database. We removed probes with expression intensity that did not exceed background noise in at least 50% of all tissue samples. We chose the probe with the highest differential stability among donors and scaled robust sigmoid to normalise expression data. These processing procedures generated normalised expression data of 15,633 genes for each tissue sample. We matched tissue samples to regions in the Desikan-Killiany atlas (Desikan et al., 2006) and aggregated samples within each region. Specifically, samples were averaged separately for each donor then averaged across donors.

In this study, SZ risk genes were derived from two studies of the Schizophrenia Working Group of the PGC published in 2014 and 2022. Specifically, 348 protein-coding genes based on gene annotation method obtained from the former study. 120 prioritized genes, which met their defined priority criteria, obtained from the latter study. There were 36 duplicated genes between the two gene sets, and another 98 genes were not detected in AHBA database. Consequently, a total of 334 schizophrenia risk genes were included for analysis. The two gene sets are listed in Supplemental Tables S1 and S2.

### 2.6 Data analysis

In this study, demographic and clinical characteristics of schizophrenia patients and healthy controls were compared. Differences in numerical variables were analysed using, depending on the normality distribution of data, either two-sample t-tests or Mann-Whitney U-tests. Moreover, categorical variables like sex and educational level were compared between groups using *χ*^2^ test. Using a two-tailed test, *P* < 0.05 is regarded as indicating a statistically significant difference between groups.

We applied linear regression model to investigate the regional t statistics of four cortical features across each region while adjusting for covariates of age, sex, educational level, and estimated intracranial volume (eTIV). The resulting t-map provided insights into regional variation in anatomical morphology between the SZ patients and HCs group. Such variations may represent specific changes caused by schizophrenia itself, potentially offering important clinical implications.

Partial least-squares (PLS) regression was employed to investigate potential associations between transcriptional profiles of genes and patient-control differences in anatomical morphology. The gene expression data (X) was used as predictor variables for cortical anatomical variation (Y), with t-maps of the four cortical variants serving as response variables. XPLS1 and YPLS1, which represent the first PLS components of X and Y, respectively (i.e., the first column of the scores matrix for X and Y), were extracted for correlation analysis to describe the correlation between X and Y. To evaluate whether the correlation between XPLS1 and YPLS1 explained more variance than the null hypothesis between cortical variants and genomic expression, null model based on spherical rotations, to account for spatial autocorrelation of cortical anatomical variants was used (Alexander-Bloch et al., 2018, Arnatkeviciute et al., 2019, Burt et al., 2020). Specifically, we initially generated 1,000 random spatial spin of the cortical regions to establish the null distribution of neuroimaging data. Then, the *P*_spin_ values were obtained by comparing the *P* value from empirical data to the null models by two-side permutation test (Fulcher et al., 2021, Váša et al., 2018). Meanwhile, we assessed the correlation between each gene and cortical anatomical variation based the spatial spin null model. Specifically, we obtained the empirical weight values of each gene on XPLS1 and the weight values from 1000 null models, then, the *P*_spin_ was calculated for each gene by conducting a two-sided permutation test. All *P*_spin_ values have undergone FDR correction, with corrected *P* value (*P*_DFR_) of <0.025 considered to indicate statistical significance.

To investigate the potential impact of sex on research results, we replicated the above-mentioned analysis process using male samples only. Specifically, we restricted our data samples to male participants, and used a subset of the AHBA dataset consisting of only five male donors.

Variable importance in projection (VIP) scores were calculated for four structural indices to assess their contribution to the model. VIP scores are a measure of variable importance in multivariate data analysis, providing information on how much a variable contributes to explaining the variation in the response variables (Chong and Jun, 2005). The variables with VIP scores greater than 1 were considered significant in predicting the PLS regression model.

Lastly, partial correlation analysis was employed to explore the relationship between scores on two neuropsychological tests (PANSS and MCCB) and four anatomical indicators (CT, CA, CV and LGI) while controlling for covariates including age, sex, educational level, and eTIV. To minimize false positives, we utilized FDR correction and set a significance threshold of 0.05.

## 3 Results

### 3.1 Sample characteristics

Details of the sample characteristics are reported in Table 1. The two groups did not differ significantly with regards to age or sex. However, a significant difference in educational level was observed between the HC and SZ groups (*P* < 0.05), with patients showing lower levels of education.

### 3.2 Anatomical feature alterations

To obtain t-maps of four distinct anatomical features, we employed a linear regression model with covariates including age, sex, education level, and eTIV. We obtained the t statistic value for the group variable in each cortical region, revealing the differences in anatomy between SZ patients and normal subjects. Positive and negative t values reflected the presence of increased and decreased anatomical features in SZ patients relative to controls, respectively.

**Fig. 2:**
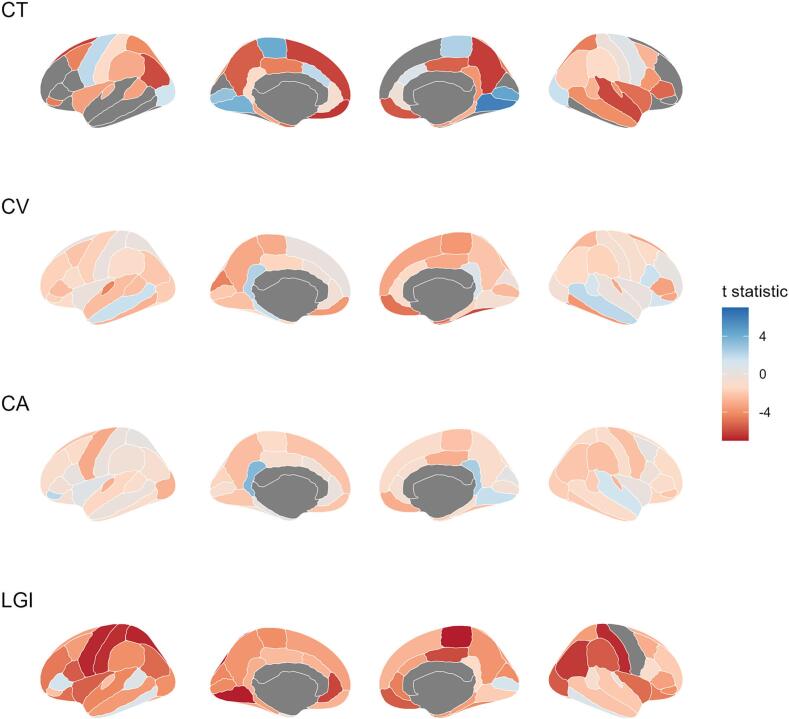
T-maps of cortical thickness (CT), cortical volume (CV), cortical area (CA) and local gyrification index (LGI) between patient with schizophrenia and healthy control groups.

**Supplementary figure 1:**
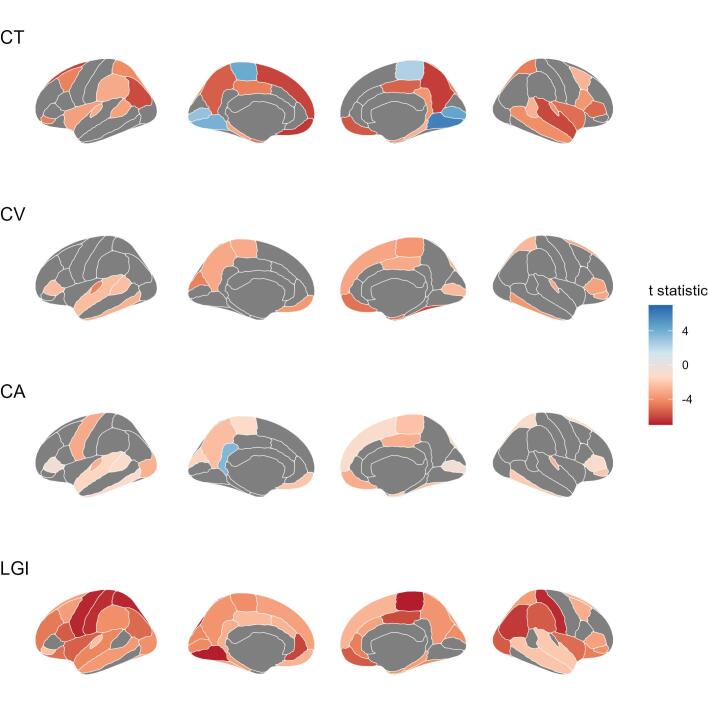


Compared to HCs, SZ patients showed significant differences in CT across 55 brain regions with an increase in eight regions after multiple comparisons correction (*P* < 0.05). Supporting details can be found in Supplemental Table S3 and illustrated in Fig. 2 **(CT)**. We observed lower CV values in 23 brain regions of SZ patients than HCs, while no significant increases were detected, as depicted by Fig. 2 **(CV)** and outlined in Supplemental Table S4. Six regions of CA showed significantly smaller values in SZ patients than HCs, while one region had an increased value (Fig. 2 **(CA)** and Supplemental Table S5). Our study also revealed significantly lower LGI values in all 55 brain regions measured for SZ patients compared to HCs, as summarized in Supplemental Table S6 and presented in Fig. 2 **(LGI)**. The results for brain regions that only had statistically significant variation are presented in Supplemental Fig. S1.

### 3.3 SZ risk genes related to cortical anatomical variation

**Fig. 3:**
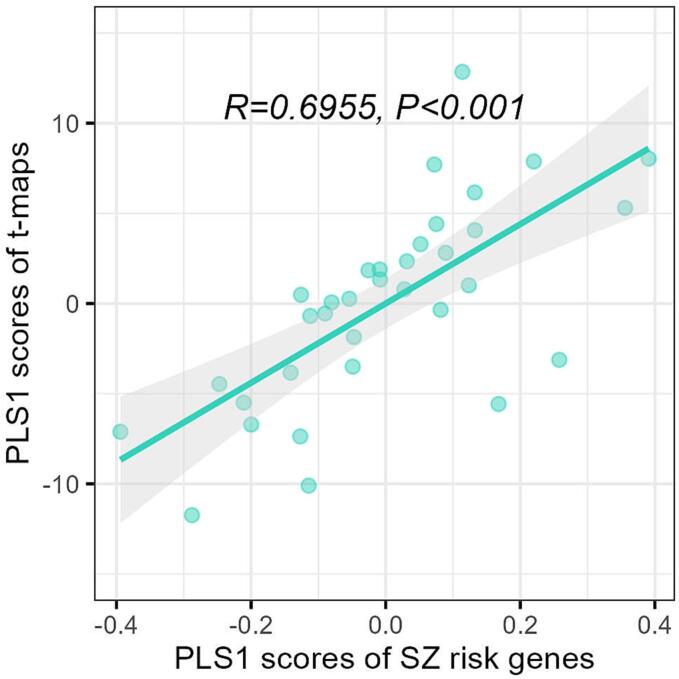
Correlation between PLS1 scores of schizophrenia risk genes and PLS1 scores of anatomical variation t-maps. *R*: spearman correlation coefficient; *P*: false discovery rate corrected *P* value*.*

In this study, we utilized PLS regression to evaluate the relationship between the variability of four anatomical characteristics in brain regions of individuals with schizophrenia and the transcription profile of SZ risk genes. Due to the AHBA dataset solely containing transcriptomic data from two right hemispheres, we employed the left hemisphere for the analysis. Therefore, the matrix of response variables Y constitutes 34 regions × 4 t-maps of four anatomical features while the matrix of predictor variables X represents 34 regions × 334 SZ risk genes. The outcomes revealed that XPLS1 and YPLS1, denoting the first PLS component of X and Y, respectively, were spatially related (r = 0.6955) with *P*_spin_ < 0.001 (Fig. 3). Further, we subsequently obtain the *P*_spin_ and *P*_FDR_ of each SZ risk gene by space spin null model. The results revealed that 4 SZ risk genes displayed a *P*_spin_ value of <0.025, of which, one gene (PSKH1) under-expression and three genes (GFOD2, KIAA1549 and RANGAP1) overexpression corresponding to the degree of cortical variation, but none of them remained significant after correcting for FDR (*P*_FDR_ >0.05). In the male subset analysis, our investigations revealed only one gene (GFOD2) with *P*_spin_ <0.025, but after FDR correction, it no longer showed statistical significance.

### 3.4 Relationship of all AHBA gene expression and cortical anatomical variation

**Fig. 4:**
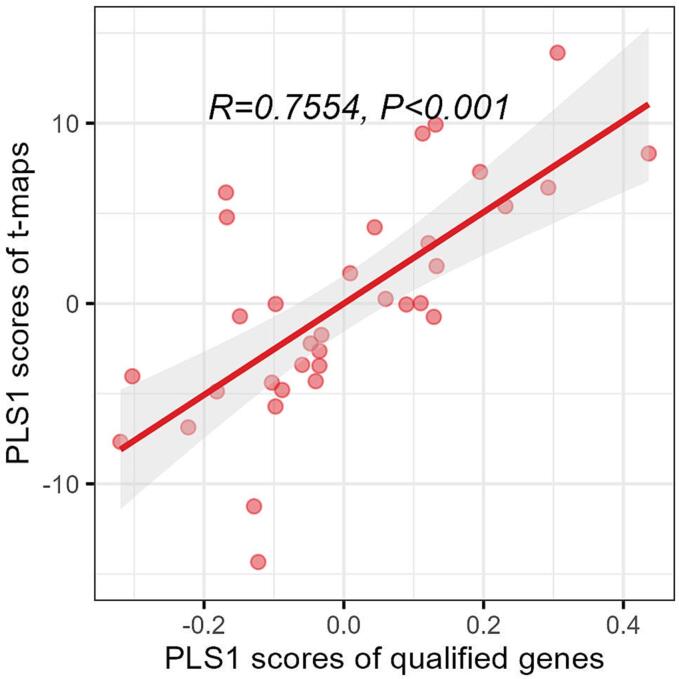
Correlation between PLS1 scores of qualified AHBA genes and PLS1 scores of anatomical variation t-maps. *R*: spearman correlation coefficient; *P*: false discovery rate corrected *P* value*.*

**Fig. 5:**
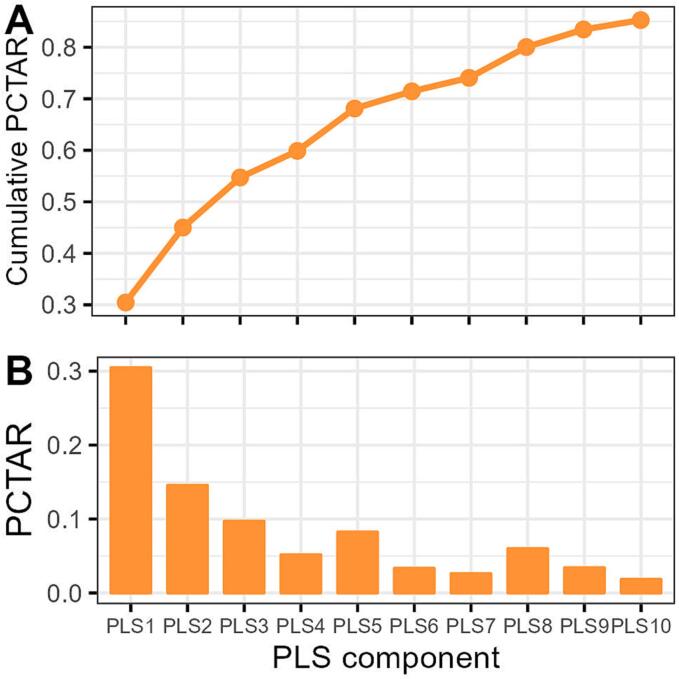
The explanation (A) of each PLS component and cumulative explanation (B) for all genetic variations.

Our analysis of cortical anatomical variation across all qualified 15,633 genes in the AHBA was conducted in a similar manner to the SZ risk gene analysis. Results showed that XPLS1 and YPLS1 were correlated with a correlation coefficient of 0.7554 (*P*_spin_ <0.001) (Fig. 4). Notably, XPLS1 was found to explain 30.46% of the variance in gene expression profiles, as depicted in Fig. 5. A total of 96 genes demonstrated significant correlations with anatomical variation (*P*_spin_ <0.025). Of these 56 genes exhibited a positive correlation between their expression and anatomical variation, while the other 40 gene show negative correlation. However, after multiple comparison correction, none of the genes remain significant. (Supplemental Table S7). Analysis of the male subset was also performed, revealing a significant correlation between XPLS1 and YPLS1 (r = 0.7650, *P*_spin_ <0.001). A total of 109 potential SZ risk genes were identified in the male subset, with 53 of these genes overlapping with those detected in the entire dataset (Supplemental Table S8).

### 3.5 The contribution of each t-map to PLS model

**Fig. 6:**
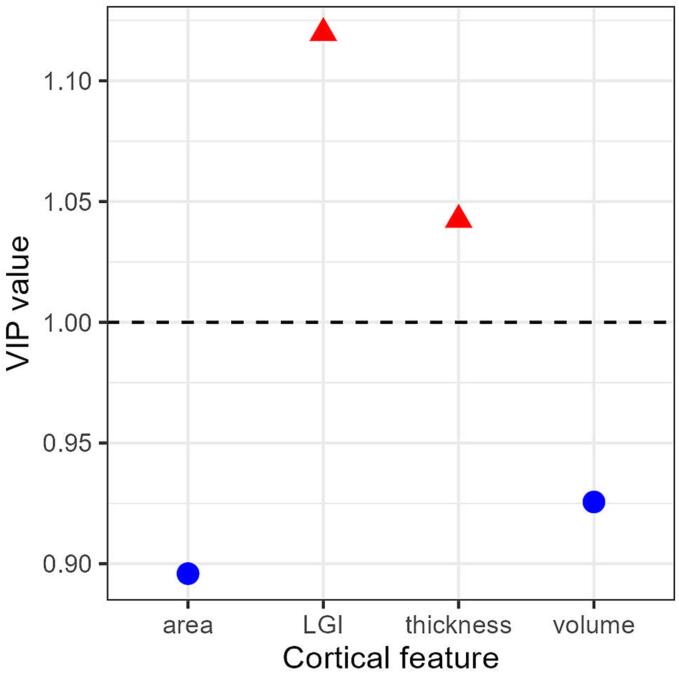
Variable importance in projection (VIP) values of t-maps for four anatomical features.

We calculated the VIP value of each t-map to represent its contribution to the PLS model, which indicates the corresponding variable contributes to explaining gene expression profiles. The results showed that LGI and CT displayed relatively greater impact compared to the other two features, possibly indicating a greater susceptibility to genetic influences (Fig. 6).

### 3.6 PANSS and MCCB correlations with morphological features

**Fig. 7:**
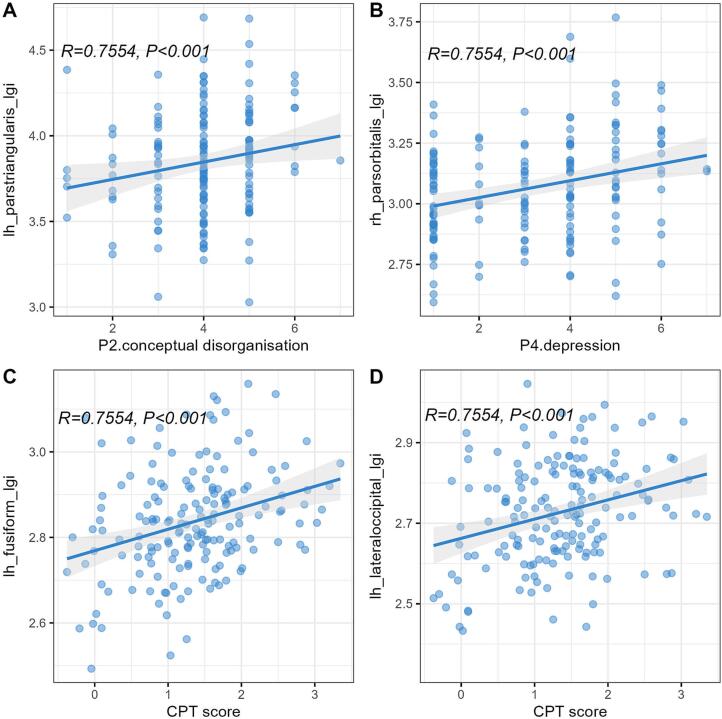
Positive association between the local gyrification index (LGI) of brain regions and individual items of the Positive and Negative Syndrome Scale (PANSS) and MATRICS Consensus Cognitive Battery (MCCB): left pars triangularis LGI with conceptual disorganisation item (P2) (A), right pars orbitalis LGI with depression item (P4) (B), Continuous Performance Test-Identical Pairs (CPT-IP) score with left fusiform LGI (C) and left lateral occipital LGI (D).

The regional correlation between the total PANSS score and subscale scores of positive, negative, and general psychopathology scores with morphological features. However, significant correlations were observed when examining individual items of the PANSS. Specifically, conceptual disorganization (P2) and excitement (P4) were significantly associated with three and one regions of the LGI, respectively, while depression (G6) was correlated with three regions of the CT. Regarding cognitive function, nine regions of the LGI were significantly associated with attention/vigilance, as measured by the Continuous Performance Test-Identical Pairs (CPT-IP), while one region of the CT showed a significant negative correlation with verbal learning as assessed by the Hopkins Verbal Learning Test – Total Recall (HVL-TR). Further details are presented in Supplemental Table S9, and partial results are illustrated via scatter plots in Fig. 7.

## 4 Discussion

The present study utilized a large sample of structural brain images to offer a comprehensive depiction of the variations in brain anatomy among patients with schizophrenia compared to healthy controls, and simultaneously explored the correlation between structural brain indicators and genetic and clinical phenotypes for the first time. We observed wide and consistent variations in brain structure in schizophrenia, which had a significant association with the expression profile of schizophrenia risk genes and the manifestation of psychotic symptoms. This study provides fresh and compelling evidence for investigating psychiatric disorders based on brain imaging, which further enriches the neurodevelopmental hypothesis.

The anatomical structures demonstrated a significant range of variation between SZ and HC individuals, as indicated by the differences observed in 55 regions of cortical thickness, 23 regions of volume, 7 regions of area, 55 regions of LGI, and 30 regions of subcortical structures (based on the DK atlas). The findings presented in this study are consistent with prior research. Takayanagi et al. (Takayanagi et al., 2019) conducted a vertex-wise thickness analysis comparing SZ and HCs groups and discovered significant cortical thinning in schizophrenia patients, mostly in frontal and temporal regions, when compared to healthy individuals. The ENIGMA Schizophrenia Working Group conducted a study including 4,474 schizophrenia patients and 5,098 healthy participants of different ethnicities and countries, which also found widespread surface area reductions in the schizophrenia group (van Erp et al., 2018). Consistent with previous studies, increased ventricular volume in subcortical structures was observed in schizophrenia patients (van Erp et al., 2016, Okasha and Madkour, 1982). A study following up on the abnormalities of cortical and subcortical structures in schizophrenia cohorts over five years revealed an increase in lateral ventricle and third ventricle volume (Nesvåg et al., 2012). These structural variants, such as cortical thinning and decreased volume, observed in schizophrenic patients, have been attributed to various factors, including the disease itself (Liu et al., 2014, Rais et al., 2012), severity (Padmanabhan et al., 2015, Walton et al., 2017), antipsychotic medication (Vita et al., 2015), and age and duration of the illness (van Erp et al., 2018).

In recent years, imaging genetics has emerged as a cross-disciplinary approach that utilizes brain imaging techniques to evaluate the structure and function of the human brain phenotype. This method enables understanding the effects of genes on behaviour or mental illness based on more objective measures of macrostructure evaluations (Morgan et al., 2019, Fornito et al., 2019). Romero-Garcia et al. found that magnetization of specific cortical regions in healthy adolescents, which is associated with schizophyte, shows colocalization with expression patterns of genes previously implicated in schizophrenia (Romero-Garcia et al., 2020). A recent study explored the relationship between CT heterogeneity in schizophrenia and inter-regional variation in neural cell types, as inferred from gene expression data and genomic variation and found CT heterogeneity in schizophrenia may be related to inter-individual variation in cell-type specific functions (Di Biase et al., 2022/04/01 2022). Another study, which used a similar research approach, found that the distribution of cortical neural cells in four major mental illnesses has similar characteristics, suggesting that they have a common brain dysfunction mechanism (Ardesch et al., n.d.).

The current investigation utilized a spatial correlation approach to explore the link between genetics and cerebral cortex anatomical variant. Remarkably, after implementing a rigorous false positive control based on the spatial autocorrelation method, no genes associated with cortical anatomical variation in schizophrenia were detected, which diverges from prior research. Sarah E. Morgan et al. identified 3,098 genes from AHBA with abnormal morphometric similarity in schizophrenia (Morgan et al., 2019). Ji et al. found that expression levels of 98 genes from AHBA showed significant cross-sample spatial correlations with CV changes in schizophrenia (Ji et al., 2021). It should be noted that the aforementioned study used PLS regression or spearman rank correlation method to identify genes related to brain imaging abnormalities in SZ, without taking into account spatial-autocorrelation as well as gene co-expression within the gene set, which may raise false-positive results (Fulcher et al., 2021, Wei et al., 2022). In addition, although genetic factors play an important role in the onset of SZ, involving hundreds of genes, no definitive pathogenic gene that is completely related to SZ has been identified. Therefore, the effect of each gene on the onset of SZ is very weak, which may also be the reason why this study failed to find genes with statistical significance.

Examining the relationship between symptomatology, cognitive function, and cortical morphology may allow for the identification of the significance of anatomical variants of the cerebral cortex in SZ. There is compelling evidence that patients with SZ experience a reduction in executive function, working memory, attention, and situational memory, which is linked to volume and cortical thickness in multiple brain regions (Gur et al., 2000, Nestor et al., 2002, Seidman et al., 1994, Zipparo et al., 2008), particularity the frontal cortex and temporal lobe (Zipparo et al., 2008, Ehrlich et al., 2012). Our study produced significant results for CT and LGI in relation to symptomatology and cognitive function across several brain areas. Notably, most of the brain regions connected to psychiatric symptoms are located in the inferior frontal gyrus, while CPT-IP of MCCB was associated with LGI in various brain regions, including the temporal, parietal, and occipital lobes.

It is noteworthy that this study found both CT and LGI exhibited more prominent roles in both the correlation with genes and clinical phenotypes, while CA and CV were less significant. CV is defined as the product of CT and CA. The cerebral cortex is modularly organized into ontogenetic columns perpendicular to the surface of the brain (Mountcastle, Apr 1997), with CT determined by the number of neurons within a column and CA determined by the number of columns (Rakic, Sep 1995). CT and CA are believed to be determined by different types of progenitor cells (Kriegstein et al., Nov 2006), with intermediate progenitor cells (IPC) amplifying each radial unit to form CT and early proliferation of radial unit progenitor cells leading to an increase in the number of ontogenetic columns and thus an increase in CA (Pontious et al., 2008). This study reveals a stronger association of CT variants with SZ, providing the basis for further investigation of the corresponding cellular and genetic mechanisms underlying CT. Cortical gyrification, represented by LGI, is thought to reflect how the brain manages the problem of packing an increasingly large cortical surface area into a limited cranial vault (White et al., Feb 2010). LGI considered to be determined by factors that occur early in brain maturation (Razavi et al., 2015). Therefore, LGI changes may be closely related to mental disease susceptibility genes, which can reflect the susceptibility to mental disorders. In individuals with SZ, abnormal LGI has been reported in first-episode SZ, siblings of patients, and high-risk and at-risk individuals (Matsuda and Ohi, 2018), suggesting that it may be an imaging biomarker of SZ.

The current findings of the study should be interpreted in the context of its limitations. Firstly, the majority of patients included in the study were chronic schizophrenia patients who had either discontinued or were taking their medication irregularly before admission, and the impact of prior medication usage was not considered in the analyses of cortical measurements and clinical scales, potentially leading to confounding of the results. Secondly, the AHBA transcriptome database utilized in this study consisted only of healthy individuals, who differed ethnically from the present study population and had a small sample size of just six individuals in the left hemisphere of the brain, which could introduce bias into our findings. Therefore, future studies investigating the transcriptome data and imaging are required to replicate these discoveries. Thirdly, while we utilized a null spatial spin model and performed FDR for P values to enhance the reliability of our results, external validation with independent samples is still necessary in future research. Finally, our analysis was mainly exploratory, and correlation studies cannot establish causal relationships. Consequently, caution must be observed when interpreting our findings.

In conclusion, our study explored the interplay among gene expression, neuroimaging data, and disease phenotype levels in schizophrenia. We confirmed substantial structural abnormalities in certain brain regions in individuals with schizophrenia. Additionally, we identified CT and LGI associated with genes expression and clinical phenotypes. Our findings offer important insights for further investigation of the genetic mechanisms underlying these structural variations and suggest promising target brain regions for disease intervention.

## Declaration of Competing Interest

The authors declare that they have no known competing financial interests or personal relationships that could have appeared to influence the work reported in this paper.

## Data Availability

Data will be made available on request.
