# Supplementary material for: Cortical anatomical variations, gene expression profiles, and clinical phenotypes in patients with schizophrenia
Source: Neuroimage Clin. 2023 Jun 9;39:103451. doi: 10.1016/j.nicl.2023.103451 (PMC10509526; doi:10.1016/j.nicl.2023.103451)
Supplement: Supplementary data 1 [file mmc1.docx]

Supplemental Table 1. Genes extracted from the GWAS of PGC in 2014

| DPYD | CHRNA3 | VRK2 | TMEM219 | CENPM | PCGEM1 | EPHX2 |
| --- | --- | --- | --- | --- | --- | --- |
| MIR137 | CHRNA5 | ADAMTSL3 | YPEL3 | CYP2D6 | GPM6A | NLGN4X |
| ARL3 | CHRNB4 | GOLGA6L4 | CACNA1I | FAM109B | CSMD1 | RIMS1 |
| AS3MT | IREB2 | ZSCAN2 | MSL2 | NAGA | CUL3 | DFNA5 |
| C10orf32 | PSMA4 | ANKRD44 | NCK1 | NDUFA6 | MMP16 | MPP6 |
| CNNM2 | IMMP2L | BOLL | PCCB | SEPT3 | GRIN2A | OSBPL3 |
| CYP17A1 | SNX19 | COQ10B | PPP2R3A | SHISA8 | PRKD1 | MAN2A1 |
| INA | ZNF804A | HSPD1 | SLC35G2 | SMDT1 | ATXN7 | MIR548AJ2 |
| NT5C2 | CNKSR2 | HSPE1 | STAG1 | SREBF2 | C3orf49 | GALNT10 |
| PCGF6 | CACNB2 | MARS2 | GRIA1 | TCF20 | PSMD6 | C11orf87 |
| PDCD11 | LRP1 | PLCL1 | PJA1 | TNFRSF13C | THOC7 | TMTC1 |
| SFXN2 | MYO1A | RFTN2 | SGSM2 | WBP2NL | ACD | PODXL |
| TAF5 | NAB2 | SF3B1 | SMG6 | BTBD18 | C16orf86 | FAM5B |
| TRIM8 | NDUFA4L2 | CHADL | SRR | C11orf31 | CENPT | C1orf132 |
| USMG5 | NXPH4 | EP300 | TSR1 | CLP1 | CTRL | CD46 |
| WBP1L | R3HDM2 | L3MBTL2 | GRM3 | CTNND1 | DDX28 | CR1L |
| CACNA1C | SHMT2 | RANGAP1 | VPS14C | MED19 | DPEP2 | KCNB1 |
| TSNARE1 | STAC3 | KCNV1 | KDM4A | SERPING1 | DPEP3 | PTGIS |
| SLC39A8 | STAT6 | CNTN4 | PTPRF | TMX2 | DUS2L | C12orf79 |
| MAD1L1 | TAC3 | DRD2 | CILP2 | YPEL4 | EDC4 | DPP4 |
| ZSWIM6 | TMEM194A | IGSF9B | GATAD2A | ZDHHC5 | ENKD1 | SLC4A10 |
| ABCB9 | LRRIQ3 | GLT8D1 | HAPLN4 | LUZP2 | ESRP2 | NOSIP |
| ARL6IP4 | C2orf82 | GNL3 | MAU2 | DGKI | GFOD2 | PRR12 |
| C12orf65 | EFHD1 | ITIH1 | NCAN | PTN | LCAT | PRRG2 |
| CDK2AP1 | GIGYF2 | ITIH3 | NDUFA13 | TLE1 | NFATC3 | RCN3 |
| MPHOSPH9 | KCNJ13 | ITIH4 | PBX4 | AKT3 | NRN1L | RRAS |
| OGFOD2 | NGEF | MUSTN1 | SUGP1 | SDCCAG8 | NUTF2 | SCAF1 |
| PITPNM2 | ESAM | NEK4 | TM6SF2 | ANKRD63 | PARD6A | C12orf42 |
| RILPL2 | MSANTD2 | NISCH | TSSK6 | PAK6 | PLA2G15 | AC005609.1 |
| SBNO1 | NRGN | NT5DC2 | ANP32E | PLCB2 | PSKH1 | CD14 |
| SETD8 | VSIG2 | PBRM1 | APH1A | ZNF536 | PSMB10 | DND1 |
| AC073043.2 | TCF4 | SMIM4 | C1orf51 | MEF2C | RANBP10 | HARS |
| C2orf47 | AMBRA1 | SPCS1 | C1orf54 | TBC1D5 | SLC12A4 | HARS2 |
| C2orf69 | ARHGAP1 | STAB1 | CA14 | CDC25C | SLC7A6 | IK |
| TYW5 | ATG13 | TMEM110 | OTUD7B | CTNNA1 | SLC7A6OS | NDUFA2 |
| FES | CHRM4 | TMEM110-MUSTN1 | PLEKHO1 | EGR1 | THAP11 | PCDHA1 |
| FURIN | CKAP5 | ALDOA | VPS45 | ETF1 | TSNAXIP1 | PCDHA10 |
| MAN2A2 | CREB3L1 | ASPHD1 | SNAP91 | FAM53C | EPC2 | PCDHA2 |
| TRANK1 | DGKZ | C16orf92 | PLCH2 | GFRA3 | ATPAF2 | PCDHA3 |
| AL049840.1 | F2 | DOC2A | ERCC4 | HSPA9 | DRG2 | PCDHA4 |
| APOPT1 | HARBI1 | FAM57B | MLL5 | KDM3B | GID4 | PCDHA5 |
| BAG5 | MDK | GDPD3 | PUS7 | REEP2 | LRRC48 | PCDHA6 |
| CKB | ZNF408 | HIRIP3 | SRPK2 | BCL11B | MYO15A | PCDHA7 |
| KLC1 | CCDC39 | INO80E | RERE | AC005477.1 | RAI1 | PCDHA8 |
| PPP1R13B | DNAJC19 | KCTD13 | SLC45A1 | RGS6 | SREBF1 | PCDHA9 |
| TRMT61A | FXR1 | MAPK3 | ATP2A2 | HCN1 | TOM1L2 | TMCO6 |
| XRCC3 | ACTR5 | PPP4C | C4orf27 | CA8 | TLE3 | WDR55 |
| ZFYVE21 | PPP1R16B | SEZ6L2 | CLCN3 | CYP26B1 | CNOT1 | ZMAT2 |
| AC027228.1 | SLC32A1 | TAOK2 | NEK1 | GRAMD1B | SLC38A7 |  |
| AGPHD1 | FANCL | TBX6 | FUT9 | SATB2 | CLU |  |

Supplemental Table 2. Genes extracted from the GWAS of PGC in 2022

| AC068490.2 | CRHR1 | FAM114A2 | KIAA1549 | NEGR1 | RERE | THAP8 |
| --- | --- | --- | --- | --- | --- | --- |
| ACE | CSMD1 | FAM216A | KLF6 | NLGN4X | RNASEH2C | THCC7 |
| ACTR1B | CTD-2008L17.2 | FAM83D | LINC00320 | NRIP1 | RP11-10L12.4 | TMTC1 |
| AKT3 | CUL9 | FTSJ2 | LINC01068 | NXPH1 | RP11-165J3.6 | TRPC4 |
| ALMS1P | CYP7B1 | FURIN | LINC01088 | OPCML | RP11-399D6.2 | TSNARE1 |
| ASPHD1 | DARS2 | GABBR2 | LRRC4B | PAK6 | RP11-490G2.2 | TXNRD1 |
| ATG13 | DCC | GATAD2A | LSM1 | PCCB | RP11-507B12.2 | WBSCR17 |
| ATP2A2 | DCLK3 | GPM6A | MAD1L1 | PCDHA2 | RP11-53O19.3 | WDR76 |
| BCL11B | DDHD2 | GPR98 | MAN2A1 | PCDHAB | RP11-586K2.1 | WSCD2 |
| BCL2L12 | DLGAP2 | GRAMD1B | MAPK3 | PCGF3 | RP11-73M18.2 | ZNF804A |
| BNIP3L | DNAJA3 | GRIN2A | MAPT | PONXL3 | SETD6 | ZNF823 |
| C12orf43 | DPYD | GRM1 | MLXIP | PDE4B | SF3B1 | ZNF835 |
| CACNA1C | EMB | HYI | MOB4 | PDIA3 | SGCD |  |
| CALN1 | EMX1 | IL1RAPL1 | MSI2 | PJA1 | SLC39AB |  |
| CISD2 | ENOX1 | IMMP2L | MY019 | PLCH2 | SLC4A10 |  |
| CLCN3 | ENSG00000262319 | INO80E | NAB2 | PSMA4 | SLC9B1 |  |
| CNTN4 | EPN2 | IRF3 | NDFIP2 | PTPRD | SNAP91 |  |
| CREB3L4 | EYS | KANSL1 | NEBL | R3HDM2 | SP4 |  |
