# Supplementary material for: Cortical anatomical variations, gene expression profiles, and clinical phenotypes in patients with schizophrenia
Source: Neuroimage Clin. 2023 Jun 9;39:103451. doi: 10.1016/j.nicl.2023.103451 (PMC10509526; doi:10.1016/j.nicl.2023.103451)
Supplement: Supplementary data 8 [file mmc8.docx]

**Supplemental Table 9.** Partial correlations between cerebral morphological features and symptomatology indicators

| Symptomatology | Features | Brain regions | *N*^a^ | Partial *R*^b^ | 95%CI | *P value* | Corrected *P value* |
| --- | --- | --- | --- | --- | --- | --- | --- |
| PANSS |  |  |  |  |  |  |  |
| P2. Conceptual disorganisation | LGI | Left pars triangularis | 159 | 0.314087 | (0.16, 0.45) | 0.000069 | 0.004692 |
| P2. Conceptual disorganisation | LGI | Right pars triangularis | 158 | 0.257551 | (0.10, 0.40) | 0.001261 | 0.042874 |
| P2. Conceptual disorganisation | LGI | Right pars orbitalis | 158 | 0.245530 | (0.09, 0.39) | 0.002146 | 0.048643 |
| P4. Excitement | LGI | Right pars orbitalis | 158 | 0.284020 | (0.13, 0.42) | 0.000357 | 0.024276 |
| G6. Depression | CT | Left lateral orbitofrontal cortex | 161 | -0.286668 | (-0.42, -0.14) | 0.000273 | 0.009282 |
| G6. Depression | CT | Left pars orbitalis | 161 | -0.243864 | (-0.39, -0.09) | 0.002085 | 0.047260 |
| G6. Depression | CT | Left pars triangularis | 161 | -0.292841 | (-0.43, -0.14) | 0.000198 | 0.009282 |
| MCCB |  |  |  |  |  |  |  |
| CPT-IP | LGI | Left fusiform gyrus | 166 | 0.269661 | (0.12, 0.41) | 0.00052 | 0.017680 |
| CPT-IP | LGI | Left lateral occipital cortex | 166 | 0.221485 | (0.07, 0.36) | 0.004619 | 0.034899 |
| CPT-IP | LGI | Left parahippocampal gyrus | 166 | 0.255175 | (0.11, 0.39) | 0.001049 | 0.017833 |
| CPT-IP | LGI | Left pars opercularis | 166 | 0.239534 | (0.09, 0.38) | 0.002141 | 0.024265 |
| CPT-IP | LGI | Left rostral middle frontal cortex | 166 | 0.247638 | (0.10, 0.39) | 0.001487 | 0.020223 |
| CPT-IP | LGI | Right fusiform gyrus | 165 | 0.227329 | (0.08, 0.37) | 0.003731 | 0.034899 |
| CPT-IP | LGI | Right parahippocampal gyrus | 165 | 0.259214 | (0.11, 0.40) | 0.000899 | 0.017833 |
| CPT-IP | LGI | Right pars triangularis | 165 | 0.27887 | (0.13, 0.42) | 0.000341 | 0.017680 |
| CPT-IP | LGI | Right rostral middle frontal gyrus | 165 | 0.223084 | (0.07, 0.37) | 0.004448 | 0.034899 |
| HVLT-R | CT | Right rostral middle frontal gyrus | 188 | -0.26179 | (-0.39, -0.12) | 0.000331 | 0.022508 |

*Note*: PANSS = Positive and Negative Syndrome Scale; MCCB = MATRICS Consensus Cognitive Battery; CPT-IP *=* Continuous Performance Test-Identical Pairs; HVLT-R = Hopkins Verbal Learning Test-Revised; *N* = Patient number; LGI = Local gyrification index; CT = Cortex thickness.

^a^ Different N are due to missing values on one or more tests for some subjects.

^b^ The cerebral morphological features linked with individual items of the PANSS were subjected to Spearmen correlation, individual items of the MCCB were subjected to Pearson correlation, and partial R was calculated by adjusting for age, sex, educational level, and eTIV.
